# Innovation through telemedicine to improve medication abortion access in primary health centers: findings from a pilot study in Musanze District, Rwanda

**DOI:** 10.1186/s12889-025-22629-z

**Published:** 2025-05-07

**Authors:** Ndola Prata, Karen Weidert, Evangeline Dushimeyesu, Eugène Kanyamanza, Dushimiyimana Blaise, Sharon Umutesi, Eugène Ngoga, Felix Sayinzoga

**Affiliations:** 1 Bixby Center for Population, Health and Sustainability, School of Public Health, University of California, Berkeley, USA; 2 Rwanda Health Initiative for Youth and Women, Kigali, Rwanda; 3 Rwanda Biomedical Center, Kigali, Rwanda; 4 Ruhengeri District Hospital, Kigali, Rwanda; 5 Rwanda Society of Obstetricians and Gynecologists, Kigali, Rwanda

**Keywords:** Telemedicine, Medication abortion, Telehealth, Sexual and reproductive health, Rwanda

## Abstract

In 2012 Rwanda expanded legal grounds for abortion to include cases of rape, incest, forced marriage, the health of a pregnant person or fetus at risk, and for minors on request in 2018. The penal code limits abortion care to doctors in hospitals, impeding access for many women. We tested an intervention that provides first-trimester medication abortion at primary health centers, using telemedicine to connect nurses/midwives to doctors in district hospitals for authorization of services. We implemented a 15-month prospective study to assess the feasibility, effectiveness, safety, and client acceptability of a hybrid telemedicine model. In the model tested, doctors provided clinical guidance by reviewing client data and lab results, and authorized the procedure via telemedicine, while the nurses/midwives consulted with the client, provided medication at the health center, and conducted follow-up over the phone or in person. Service data record forms were completed using the REDCap online platform and client exit interviews were conducted after completion of the abortion. During implementation, 242 clients received medication abortion at the health centers, with 50% of clients interviewed during client exit interviews. The protocol ensured high adherence rates; 96% completed abortion. Post-procedure complications were rare (3%) and were largely managed at health centers with remote support from a medical doctor. Vaginal bleeding (36%) and abdominal pain (41%) were the prevalent side effects experienced by clients; only 10% of clients who reported side effects needed to see a provider for management. Overall client satisfaction with services was very high (98%) and the perceived quality of services was also very high (97–99%). We conclude that this hybrid telemedicine model for the provision of first-trimester medication abortion is feasible, effective, safe and accepted by clients. Results from this study will enable revisions to the abortion clinical guidelines to include task-sharing with mid-level providers, such as nurses and midwives, via telemedicine in health centers.

**Supplementary Information:**

The online version contains supplementary material available at 10.1186/s12889-025-22629-z.

## Background

In 2012, Rwanda expanded legal grounds for abortion to include cases of rape, incest, forced marriage, and pregnancies that put the health of the pregnant person or the health of the fetus at risk [1]. In 2018, the law was expanded such that there is no criminal liability if the pregnant person is a minor [2]. However, implementation of the revised penal code limits authorization of abortion care to medical doctors in hospitals and polyclinics(medical doctor- supported clinics with beds available for hospitalization and services available 24/7), which has resulted in unequal access among Rwandan women, with often the most vulnerable women, especially those living in rural communities far from hospitals, excluded from services. Model-based estimates from 2015–2019 indicate that 54% of pregnancies in Rwanda were unintended and among the unintended pregnancies, 29% ended in abortion [3]. A study assessing causes of maternal mortality from 2017–2019 in Rwanda found abortion accounts for 8% of maternal deaths [4]; and despite a significant decline over the last two decades, the maternal mortality ratio is high, estimated at 203 in the 2019/2020 Demographic Health Survey (DHS) [5].

For Rwanda to widely increase access to abortion services and minimize barriers that encourage women to seek out unsafe alternatives, a service delivery model that ensures healthcare staff are trained and willing to provide abortion services in health centers is prudent. The use of mifepristone and misoprostol for medication abortion is a globally endorsed method, and is increasingly used worldwide [6–10]. In 2015, the World Health Organization’s (WHO) safe abortion guidance recommended that abortion services be provided at the lowest appropriate level of the healthcare system, including medication abortion up to 12 completed weeks of pregnancy. The guidelines state that mid-level health workers, including midwives, nurse practitioners, clinical officers, physician assistants, and others, can be trained to provide early abortion without compromising safety [11]. A systematic review of early abortion services in primary care in low- and middle-income countries showed that providing early medication abortion in primary care services is safe and feasible and task-shifting to mid-level providers can effectively replace doctors in providing abortion [12].

The WHO has recommended the option of telemedicine as an alternative to in-person interactions with the health worker to deliver medication abortion services in whole or in part [13] and this option has become increasingly popular among women who have the choice. The WHO defines telemedicine as the provision of health care at a distance through technology [14]. Many companies in Africa are also using telemedicine to transform access to healthcare [15]. Telemedicine can provide a unique opportunity to increase access to abortion care by maximizing existing technologies and healthcare infrastructure. In South Africa, a telemedicine model with asynchronous online consultation and instruction for home medication abortion, with uterine palpation as the only in-person component, was non-inferior to standard care when rates of abortion completion were compared. The research team also found the telemedicine model did not affect safety, adherence, or satisfaction with services [16]. Marie Stopes Ghana also launched a pilot project to understand the feasibility and acceptability of providing early medication abortion through telemedicine given abortion access constraints for Ghanian women. The telemedicine model consisted of a comprehensive telephone consultation with a qualified healthcare provider. Following the consultation, patients could choose to receive abortion medication via courier service or pick it up in person at a designated clinic. The study demonstrated how telemedicine provides access to patients with limited other options and meets patient needs surrounding discretion, convenience and timing [17].

Rwanda is at the forefront of using technology to improve general healthcare delivery in the region. With their Digital Health Strategic Plan 2018–2023, the government set an overarching goal to improve health service delivery and accessibility through digital health [18]. The Ministry of Health has prioritized the integration of digital technologies into healthcare operations, including telemedicine. Rwanda has also been acknowledged for its efforts to create a National Health Information Exchange (HIE) platform to provide a secure way for data to be shared within the health management information system (HMIS) [19]. Despite the absence of specific legal provisions for telemedicine abortion in Rwanda, certain in-person requirements pose challenges. A hybrid model, integrating in-person care with telemedicine and digital tools, presents a reasonable solution by ensuring compliance with the country's regulations for medical doctor authorization and the requirement for mifepristone administration within healthcare facilities.

For this research, we implemented a hybrid telemedicine model. Nurses and midwives at health centers conducted in-person consultations with women seeking abortion services. These healthcare providers were remotely connected to medical doctors in hospitals via telemedicine for authorization of first-trimester medication abortions. By integrating telemedicine components into in-person care, the intervention sought to improve access to abortion care. This model enabled doctors to provide remote guidance and authorization for medication abortions, ensuring compliance with Rwandan law while bringing care closer to women's homes. Existing evidence demonstrates that distance to services is a barrier and that bringing services closer to women increases utilization [20, 21]. Access at health centers allows for earlier abortion care and decreases delays in receiving services given reductions in travel time and referrals. This strategy also reduces the costs of service provision and addresses the shortage of medical doctors authorized to provide abortions in health centers in Rwanda.

The purpose of this paper is to provide evidence on the feasibility, safety, effectiveness, and acceptability of a telemedicine service delivery model implemented in Musanze, Rwanda over 15 months (from October 15, 2021 – January 11, 2023). Results from this study will support revisions to the abortion clinical guidelines and protocols to include telemedicine, utilize nurses/midwives in health centers, and include the mifepristone/misoprostol combination pack as essential medicine at the health center level in Rwanda. This study aims to contribute to the growing body of evidence from middle- and low-income countries demonstrating the successful implementation of telemedicine models that address local needs, resource limitations, and government regulations.

## Methods

### Study design and population

A 15-month prospective study was implemented to assess the feasibility, effectiveness, safety, and client acceptability of a hybrid telemedicine service delivery intervention to increase access to first-trimester medication abortion. The project was implemented in 7 health centers in Musanze District in the Northern Province by the Rwanda Health Initiative for Youth and Women (RHIYW) in collaboration with the Rwanda Biomedical Center, Rwanda Society of Obstetricians and Gynecologists, and the Bixby Center for Population, Health and Sustainability at the University of California, Berkeley. There were 16 eligible public health centers at the time this research was conducted, with 3 having religious affiliation and not eligible for participation. We selected 7 health centers for this study based on their high volume of post-abortion care (PAC) cases from January 2020 to December 2020 and their geographic location within the district. The selection criteria focused on including health centers with consistently high PAC volumes coming from rural and peri-urban areas. This approach provides both volume-specific insights and district-wide geographic representation. We assumed that facilities with high PAC cases were serving populations with higher demands for pregnancy terminations, given that the PAC cases were from the facility population catchment areas.

In Musanze, 79% of households have a mobile phone and 90% have health insurance [22]. Women in Musanze have more children than they desire—with an observed total fertility rate (TFR) of 3.5 compared to a wanted TFR of 2.9. Among women aged 25–49 years, the median age of first marriage and first birth are 22.4 years and 22.7 years, respectively. Six percent of adolescent women (15–19 years) in Musanze had begun childbearing in the most recent Demographic and Health Survey at that time (2019–20). Nearly 23% of women in the Northern Province reported sexual violence in 2019–20 [22].

All women of reproductive age (15–49 years) in Musanze seeking first-trimester medication abortion within the Rwandan legal framework were eligible to participate in the study. In Rwanda, abortion is legal under specific circumstances as outlined in the country's legal framework: risk to the woman’s life or health; rape, incest, or forced marriage; and fetal impairment [1].

### Protocol

We selected two nurses/midwives from each of the 7 participating health centers to be trained in the protocol. The research team-oriented community health workers affiliated with participating health centers to the project and trained them to make referrals. Health center staff attended project orientations and participated in abortion values clarification and attitudes transformation (VCAT) workshops.

All eligible and consenting clients requesting first-trimester medication abortion services at health centers participated in a joint consultation with a health center nurse/midwife and an authorized doctor at the district hospital who connected with the nurse/midwife via telemedicine. Before the nurses/midwives scheduled telemedicine consultation with the doctor, they verified legal eligibility and age of the pregnancy (less than 13 weeks); conducted a clinical assessment and ultrasound exam; provided pre-procedure counseling; requested laboratory tests; and entered all clinical information in an electronic patient file which could be accessed simultaneously by the nurse/midwife and the doctor in different locations. After a medical decision by the doctor, the nurse/midwife provided the misoprostol-mifepristone combination tablets and followed the approved clinical protocol to manage the case.

The clinical procedures for medication abortion were adapted from Rwanda’s Safe Abortion Guidelines developed in 2019 [2]. Medication was provided by the nurse/midwife with 200 mg of mifepristone taken orally at the health center, given the facility-based administration of mifepristone requirement. The woman was discharged with two 800 mcg doses of misoprostol to self-administer at home. The first dose of 800 mcg of misoprostol was used 24 h after mifepristone and the second dose three hours after the first dose of misoprostol. Before the client left the clinic, the nurse/midwife provided the post-procedure counseling; including how and when to take the misoprostol, what to expect after taking misoprostol (i.e. length and amount of bleeding), the danger signs to look for during the process, and when to contact a medical provider immediately (including heavy bleeding, excessive pain and fever). Additionally, the client was given a phone number for the nurse/midwife and encouraged to call regarding any issue with treatment. The nurse/midwife also provided self-care counseling, instructions for hygiene, and information about when to resume sexual activity, as well as contraceptive counseling. All of the contraceptive methods, except the IUD, could be provided during the initial consultation for medication abortion. Women were provided with their method of choice.

The nurse/midwife conducted three follow-up consultations over the telephone using a questionnaire guide. If a patient couldn't access a phone, she was asked to come in for an in-person follow-up. Follow-up took place at the following intervals: 48 h after mifepristone intake to confirm the client took the misoprostol correctly, bleeding started, and determine whether side-effects were well managed with no danger signs; 7 days after the medication abortion to ensure no complications and abortion is completing; and 14 days after medication abortion to assess completion of abortion, and ensure no signs of incomplete abortion or infection. The client returned to the health center if it was deemed necessary during the telephone consultations. A doctor was available to the nurse/midwife via telemedicine (phone or teleconference) to address concerns related to outcome and medication side effects during the follow-up appointments. The decision to refer the patient was made by the consulting doctor.

### Data collection & analysis

#### Service data record

Healthcare providers completed an individual service record for each woman receiving medication abortion service at the health center. Service data were collected and managed using REDCap (Research Electronic Data Capture) (Supplementary material Appendix 1), a secure web application for online databases [23, 24]. REDCap allowed for simultaneous viewing of the record by the nurse/midwife and consulting doctor in two different locations (health center and hospital). The client record was updated after each follow-up call/visit at 48 h, 7 days and 14 days after mifepristone was taken at the health center. The form included 7 sections, including the following: i) client identification; ii) medical history and sociodemographic information; iii) physical examination; iv) telemedicine consultation; v) medication and treatment; vi) follow-up visits; and v) treatment summary.

For monitoring purposes, a priori summaries were programmed at the beginning of the project, but any variable in the data could be assessed at any point in time. Table 1 shows the indicators used to measure the main study outcomes: feasibility, effectiveness, safety and client acceptability, and the interpretation of each of the indicators assuming successful outcomes. Data analysis for both mid-project and final reports was done using Stata version 17.0 [25].

**Table 1:** Indicators used to assess feasibility, effectiveness, safety and client acceptability of a telemedicine medication abortion service delivery model in Musanze, Rwanda

**Outcome**	**Indicator**	**Interpretation**
Feasibility	Providers complete telemedicine consultations for pregnancy termination	100% telemedicine consultations for pregnancy termination are completed by providers per protocol
	Providers demonstrate ability to dispense misoprostol-mifepristone combination following consultation	100% providers correctly dispense misoprotol-mifepristone combination per protocol
	Providers demonstrate ability to successfully provide follow-up care with remote supervision, including treatment of incomplete abortion and post abortion family planning	100% providers demonstrate ability to successfully provide follow-up care (7th day and 14th day follow-up visits in person or remotely) with remote supervision, including diagnosis and treatment of incomplete abortion and post abortion family planning; less than 5% lost to follow up
	Training and values clarifications results in provision of high quality for abortion services	100% trainees attend values clarification sessions and provide high quality services per protocol
	Supportive supervision adequate to ensure safety of medication abortion	Protocol established support supervision ensures 100% cases of correct drug administration, identification of clinical abnormalities and ectopic pregnancies during ultrasound; correct diagnosis and treatment of side effects as needed
Effectiveness	Eligible women successfully complete pregnancy termination at health center through telemedicine services	95% or higher treatment success rate for pregnancy termination at health centers through telemedicine
Safety	Appropriate management of side effects by health center provider	100% cases assessed for side effects and appropriately treated (per protocol) as needed; 100 patients are counselled on expected side effects and take home sanitary pads and pain medication
	Correct counseling and administration of the medication at health center	100% cases of correct drug use by patients
	Type and severity of complications; Incomplete cases that need additional interventions	100% cases correctly diagnosed and treated or referred per protocol, including diagnosis and treatment of continued pregnancies (less than 2%) and treatment of incomplete abortions (less than 5%)
	Number of referrals	100% of needed referred cases are done per protocol, including complications that require interventions at hospitals and requested terminations higher than 12 weeks gestation
Client Acceptability	Client satisfaction with services (both telemedicine and medication abortion at health center	90% eligible clients for pregnancy termination accept to be treated at the health center
	Client would recommend services to family and friends	90% of clients are satisfied with services at health center and would recommend services to family and friends

Indicator = description of each indicator per outcome group; Interpretation=expected level of the indicator to be considered successful

High quality services = all patients are diagnosed, treated and/or referred per protocol

Interventions that require hospital care: higher than 12 weeks gestational age; hemorrhage; pre-shock; anemia that requires blood transfusion; ectopic pregnancy; severe infections

Means, proportions and 95% confidence intervals are presented as part of the descriptive statistics for clients’ sociodemographic characteristics and outcome indicators. We use ANOVA to test for variance in pain severity and bleeding severity. We hypothesized that the variance in pain is equal to the variance in bleeding. Our hypothesis is based on the literature that vaginal bleeding is often associated with pelvic pain in various conditions, including infections, pregnancy complications, and other gynecological issues. Because both symptoms are present during abortion, we believe that together they operate similarly to other gynecological conditions. [26–28] Statistical significance was established at *p*-value < 0.05.

#### Client exit interview

Clients who received medication abortion services at participating health centers were asked by their healthcare provider to participate in an exit interview 2–3 weeks after receiving services. Only those clients who accepted to be in the study, received services at the health center and agreed to be re-contacted by the local research team were eligible for the client exit interview. Among 242 clients enrolled in the study, 236 consented to follow-up for the client exit interviews and the interviews took place continuously over the 15-month implementation period, with a random sample called to reach ~ 50% of the consented sample which was based on the availability of interviewers. Client exit interviews were conducted over the phone using a questionnaire (Supplementary material Appendix 2) to evaluate the overall acceptability and patient satisfaction with the project. Respondents were surveyed about their entire abortion experience, from initial consultation to medication administration, and post-procedure follow-up. Participants were also interviewed about their perceived quality of care, encompassing counseling, follow-up support, and provider interactions. The interview was conducted by a researcher not involved in direct service provision at the health centers. Client exit interview data were recorded in Qualtrics [29] while conducting the survey. Qualtrics automatically analyzed the results; however, researchers exported the data for further customization and analysis.

## Results

Project health centers began providing first-trimester medication abortion services on October 15, 2021. Implementation and data collection continued at health centers and hospitals until January 11, 2023. The following analyses are based on 15 months of Service Delivery Form and Client Exit Interviews. The Service Delivery Form includes information for 242 women seeking safe abortion services at project health centers and health posts, of whom 50% participated in an exit interview.

Fifty percent of the women seeking abortion were adolescents and young adults (15–24 years old); 62% had secondary or higher education; and 66% were never married (Table 2).

**Table 2:** Sociodemographic characteristics of clients who received medication abortion at 7 health centers in the Musanze District

**Variables (n=242)**		**Number**	**Percentage**	**95% CI**
**Age**				
15-19		30	12.4%	8.5 -17.2
20-24		90	37.2%	31.1 - 43.6
25-29		53	21.9%	16.9 - 27.6
30-34		40	16.5%	12.1 - 21.8
35+		28	11.6%	7.8 - 16.3
Missing		1	0.4%	0.01 - 2.3
**Education**				
No education		16	6.6%	3.8 - 10.5
Primary		74	30.6%	24.8 - 36.8
Secondary		117	48.3%	41.9 - 54.8
Above secondary		34	14.0%	9.9 - 19.1
Missing		1	0.4%	0.01 - 2.3
**Marital Status**				
Never married		159	65.7%	59.4 - 71.7
Married		40	16.5%	12.1 - 21.8
Divorced/Separated		39	16.1%	11.7 - 21.4
Missing		4	1.7%	0.5 - 4.2

These are clients treated and followed up at the health centers

As shown in Table 3, slightly more than half (53%) of the women were pregnant for the first time, but 28% had been pregnant 3 or more times. While 53% of women did not have children, among those who had the mean number of children was 2 and the vast majority of women (97%) were having their first abortion. Most women were not using contraception before the pregnancy they sought to terminate (69%).

**Table 3:** Reproductive history and family planning use among clients who received medication abortion services at 7 health centers in the Musanze District

**Variables (n=242)**		**Number**	**Percentage**	**95% CI**
**Gravida**				
1		129	53.3%	46.8 - 59.7
2		46	19.0%	14.3 - 24.5
3		29	12.0%	8.2 - 16.8
4		14	5.8%	3.2 - 9.5
≥5		24	9.9%	6.5 - 14.4
**Parity**				
0		127	52.5%	46.0 - 59.0
1		48	19.8%	15.0 - 25.4
2		31	12.8%	8.9 - 17.7
3		14	5.8%	3.2 - 9.5
4		16	6.6%	3.8 - 10.5
≥5		6	2.5%	0.9 - 5.3
**Prior Abortions**				
0		235	97.1%	94.1 - 98.8
1		6	2.5%	0.9 - 5.3
2+		1	0.4%	0.01 - 2.3
**Contraceptive use prior to this pregnancy**				
None		168	69.4%	63.2 - 75.2
Yes, regularly		9	3.7%	1.7 - 6.9
Yes, but not regularly		47	19.4%	14.6 - 25.0
Missing		18	7.4%	4.5 - 11.5

Missing values represent those who were not asked or did not want to answer

### Feasibility

All nurses/midwives completed the training that included: comprehensive abortion care; values clarification and attitudes transformation (VCAT); use of ultrasound in obstetrics; study procedures (enrollment, informed consent); telemedicine protocol; clinical procedures (clinical assessment, eligibility assessment, clinical protocol, case management, counseling, patient follow-up); and data collection using an electronic patient record. This study validates the training program for local providers, demonstrating its safety and effectiveness.

All providers demonstrated their ability to follow the study protocol including: conducting telemedicine consultation with the district doctor; performing ultrasound exams; client assessment; pre-abortion counseling; dispensing mifepristone-misoprostol combination therapy; patient follow-up, referrals, treatment of incomplete abortion and post-abortion family planning.

All health centers were appropriately equipped with laptops, internet connection and air time, making teleconsultation a success and an example to follow. The providers in the project were connected using an information and technology platform including direct phone calls, phone messaging using WhatsApp closed channels for immediate communication, scheduling teleconsultation, and discussing client treatment outcomes. Telemedicine consultation between the doctor and nurse/midwife was used with 283 patients of which 249 required abortion-related services; most teleconsultations (88%) were first consultations that lead to abortion including all of the ones eligible for services at the health center (*N* = 242); follow-up telemedicine consultation was required in less than 5% of the cases, and 7% of the teleconsultations were allocated to general patient care (Table 4).

**Table 4:** Reasons for telemedicine consultation between doctor and nurse/midwife among all reproductive health clients at 7 health centers in the Musanze District (*N*= 283)

**Reason for telemedicine consultation**	**Number of consultations**	**%**	**95% CI**
First consultation for abortion	249	88.0	83.6 - 91.5
Follow up consultation for medication abortion	13	4.6	2.5 - 7.7
Management of medication abortion complications	1	0.4	0.009 - 2.0
General patient care	20	7.1	4.4 - 10.7
Total	283	100	

### Effectiveness

All eligible women receiving services at health centers received correct treatment with 96.3% achieving abortion completion (Table 5). Hospital referrals before abortion provision were made for second-trimester abortion services, gender-based violence in need of legal support, ectopic pregnancies, ultrasound abnormalities and other medical reasons, demonstrating the high quality of services and careful protocol implementation by nurses/ midwives. Due to the strict protocol for patient follow-up at 48 h, 7 days and 14 days, only 2 cases of continued pregnancy were identified and treated accordingly and 6 cases of incomplete abortion were treated with an additional dose of misoprostol and other medications as needed (i.e. antibiotics, pain relief).

**Table 5:** Service delivery outcomes among abortion clients served at 7 health centers in the Musanze District

**N=277 **	**Number**	**%**	**95% CI**
**Referred cases to hospital**	**35**	**13.1%**	**9.0 - 17.1**
Second trimester services	16	45.7%	28.8 - 63.4
Gender based violence services	4	11.4%	3.2 - 26.7
Supply chain issues (i.e. no reactive for required blood testing)	1	2.9%	0.07 - 14.9
Trained provider not available	2	5.7%	0.7 - 19.2
Clinical observations (i.e. uterine scarring, low platelets, Rh-)	3	8.6%	1.8 - 23.1
Ectopic pregnancy	2	5.7%	0.7 - 19.2
Ultrasound abnormalities*	7	20.0%	8.4 - 36.9
**Total cases eligible for abortion at health centers**	**242**	**90%**	**82.9 - 91.0**
Abortion successfully completed	233	96.3%	93.1 - 98.3
Lost to follow-up	1	0.4%	0.01 - 2.3
Abortion failure (continued pregnancy required additional treatment)	2	0.8%	0.1 - 3.0
Treatment of incomplete abortion	6	2.5%	0.9 - 5.3

*Abnormalities included: fetal abnormalities, molar pregnancy, missed abortion, blighted ovum

Around 97% of all patients received counseling for family planning and the vast majority adopted a method. However, 39% decided to practice natural family planning. Among those who adopted modern methods, injectables were the most commonly adopted followed by implants (data not shown).

### Safety

The providers correctly implemented the clinical protocol excluding non-eligible clients, such as women with major underlying health problems that should be treated at the hospital. The ultrasound screening effectively identified ectopic pregnancies and other abnormalities (Table 5).

All clients undergoing medication abortion (242) received a dose of mifepristone at the health center and were followed up 48 h later with a phone call to ensure misoprostol was taken and determine the presence of any side effects that needed additional interventions. Only one patient could not be followed up (Table 5), demonstrating the success of the implementation of the study protocol.

Clients were counseled on what to expect related to medication side effects and could at any time contact the provider. Nausea, vomiting and shivering are well-known side effects associated with misoprostol. Pain and bleeding are also expected but can be managed with medication so women can be comfortable. Pain medication and sanitary pads were provided to all women. As shown in Table 6, vaginal bleeding (36%) and abdominal pain (41%) were the most common symptoms reported. Not surprisingly, most medication taken was for abdominal pain. All patients took pain medication home to take as recommended in case of pain. Among the clients who reported abdominal pain as a side effect, only 25% reported taking medication for pain during follow-up calls with the nurse/midwife. Just 10% of clients needed to see a provider due to side effects (Table 6).

**Table 6:** Clinical side effects and management among abortion clients served at 7 health centers in the Musanze District

**N=242 clients**									
**Symptoms**	**Number Cases**	**% Cases**	**95% CI**	**Number took medication**	**% took medication**	**95% CI**	**Number saw a provider**	**% saw a provider**	**95% CI**
Nausea	9	3.7	1.7 - 6.9	2	0.8	0.1 - 3.0	0	0.0	0.0 - 0.0
Vomiting	5	2.1	0.7 - 4.8	1	0.4	0.01 - 2.3	0	0.0	0.0 - 0.1
Shivering	4	1.7	0.5 - 4.2	0	0.0	0.0 - 0.0	2	0.8	0.1 - 3.0
Vaginal bleeding (more than expected)	87	36.0	29.9 - 24.3	17	7.0	4.1 - 11.0	8	3.3	1.4 - 6.4
Abdominal pain (more than expected)	98	40.5	34.3 - 47.0	61	25.2	19.9 - 31.2	13	5.4	2.9 - 9.0
**Total **	**203**	**83.9**	**78.6 - 88.3**	**81**	**33.5**	**27.6 - 39.8**	**23**	**9.5**	6.1 - 13.9

Note: Nausea, vomiting and shivering were self-contained after medication, no longer present at first week follow up; more than expected vaginal bleeding and abdominal pain were per client report; most self-medicated instructions received during first visit; small % saw a provider for additional instructions

Table 7 presents results from a one-way analysis of variance. We hypothesized that the variance in pain is equal to the variance in bleeding. Results demonstrated that among our study population, there was a significant difference between pain and bleeding (*p*-value for *F* = < 0.0001). Furthermore, Bartlett’s test for equal variances is statistically significant (*p*-value 0.001) thus rejecting our initial hypothesis that both variances (pain and bleeding) are equal.

**Table 7:** Results analysis of variance between pain and bleeding side effects

	Analysis of Variance				
Source	SS	df	MS	F	*p*-value
Between groups	37.447	4	9.361	102.28	<0.0001
Within groups	6.224	68	0.091		
Total	43.67	72	0.606		

Bartlett's equal-variance test: chi2(2)= 13.6150; *p*-value= 0.001

### Client acceptability

**Fig. 1:**
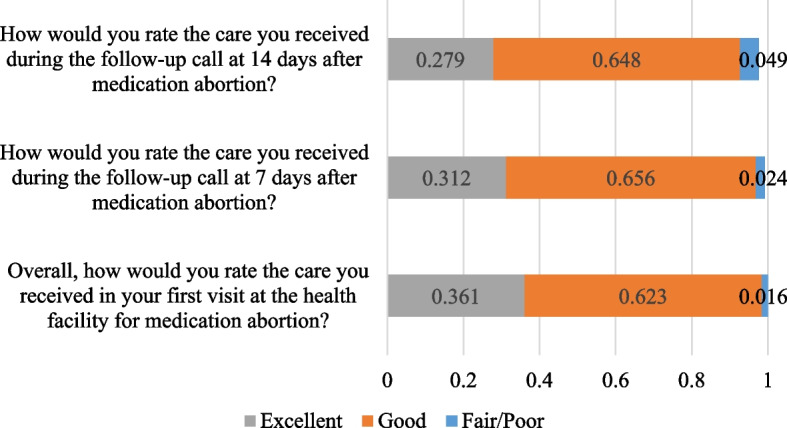
Client responses related to satisfaction with care (*N* = 122)

The majority of the interviewed clients (*N* = 122 out of 236 who consented to be contacted after discharge) rated their satisfaction with care in all three categories as excellent or good (Fig. 1).

Clients’ perception of the overall quality of care was very high. High-quality abortion care also includes quality counseling: 97.5% of respondents felt the information and explanations they received were adequate; 94.3% recalled that providers discussed sexually transmitted infections; 94.3% recalled a discussion of family planning and warning signs after initiating medication abortion (data not shown).

**Fig. 2:**
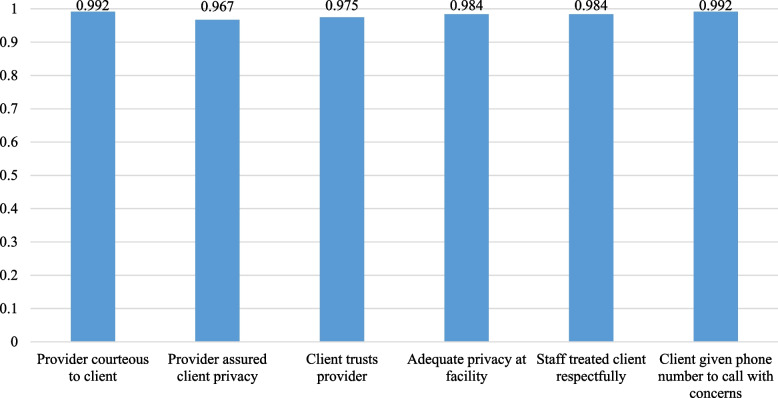
Client responses related to quality of care (*N* = 122)

As shown in Fig. 2, high-quality abortion care also includes quality counseling. Almost all (97.5%) of respondents felt the information and explanations they received were adequate (Fig. 2). Additionally, most clients recalled that providers discussed sexually transmitted infections (94.3%), family planning (94.3%) and warning signs after initiating medication abortion (94.3%) with clients (data not shown). Of the women who received medication abortion, over 93% and 95% said they would choose this method of treatment again and would recommend this treatment to a friend, respectively (data not shown).

## Discussion

This pilot project introduced first-trimester medication abortion in primary-level health centers, employing task-sharing with midlevel providers, such as nurses and midwives, to increase access to safe abortion services. We found the hybrid telemedicine model to be feasible. The nurses/midwives demonstrated their ability to follow the telemedicine protocol and clinical procedures. Nearly 90% of clients who sought an abortion were eligible for medication abortion services at the health center. As a result, task-sharing first-trimester abortion services to health center providers can reduce redundancies and delays in receiving care at hospitals. Telemedicine also creates efficiencies and reduces stress for limited healthcare workers in low-resource settings [30, 31].

In South Africa, the telemedicine model for medication abortion was designed for settings with limited resources, including poor infrastructure, limited ultrasound access, and restrictive abortion laws. To enhance resource efficiency, the model utilized an online screening format and incorporated uterine palpation as an in-person safety measure. In the Ghanaian early medication abortion study telemedicine study, 97% of the participants successfully terminated the pregnancy and 36% reported they had no other option for accessing an abortion [17]. Building on these findings, our pilot study demonstrates the potential of digital technologies to expand access to first-trimester abortion. Specifically, we provide evidence for a hybrid telemedicine model where women receive comprehensive in-person consultation at a health center, followed by remote authorization and prescription of medication from a medical doctor via telemedicine. It is important to note that all of these interventions were feasible because the infrastructure was in place to support this type of innovation. The participating health centers in our pilot study were appropriately equipped with laptops, internet and air time, making teleconsultation a success and an example to follow. Infrastructure limitations, including inadequate internet connectivity and inconsistent electricity supply, may inhibit broader replication and scalability of this model in Sub-Saharan Africa [32].

Similar hybrid telemedicine models have been successfully implemented in other resource-limited settings. In 2020, Ipas Pakistan partnered with Sehat Kahani to launch a hybrid telemedicine model to provide free contraception, safe abortion, and other gynecological services. Community health workers played a crucial role in this initiative, connecting women with online doctors and ensuring access to essential healthcare. The government helped Ipas Pakistan recruit community health workers who already have smartphones to facilitate connections with Sehat Kahani, the telehealth provider [33]. In Mexico, TeleAborto, a telemedicine abortion service, was offered at four locations: three private clinics and one community-based organization. After screening for eligibility and completing any necessary pre-abortion tests locally, participants received medication abortion packages with follow-up appointments scheduled remotely for 7 to 14 days later [34]. This study demonstrated that guided self-managed abortion through telemedicine is a safe, acceptable, and practical approach in Mexico. The researchers concluded that the model has the potential to improve access to abortion care, particularly for indigenous and rural communities, and those who rely on public health services. Like these other models, our hybrid telemedicine approach aimed to bridge the gap for women in underserved areas by connecting them with qualified healthcare providers close to home.

Our study demonstrated the telemedicine model was effective. All eligible women receiving services at health centers received correct treatment for medication abortion, including, thorough clinical assessments, accurate counseling, correct medication dosages and patient follow-up (48 hours, 7 and 14 days after procedure); treatment of incomplete abortion post-procedure, assessment of continued pregnancy cases and its management; and management of side effects when necessary. Among the 242 women who received medication abortion at the health center, 233 (96.3%) had a complete medication abortion with no complications. Women demonstrated they could safely and effectively take misoprostol tablets home, and the clinical protocol ensured high adherence to treatment. This was similar to a randomized control trial in South Africa, where home self-medication abortion did not affect adherence or safety [16].

Safety for abortion services via telemedicine in health centers was also established in our study. Nurses and midwives at health centers demonstrated that they could follow treatment and referral protocols correctly, understanding their capacity to treat clients at the health center and referring women with underlying health concerns that put them at heightened risk to higher-level facilities. However, ultrasound and other tests required before the provision of medication abortion in our hybrid model may limit access in some health centers. A systematic review found that medication abortion performed without prior pelvic examination or ultrasonogram is a safe and effective option for pregnancy termination [35]. Future telemedicine protocols may benefit from minimizing their requirement, particularly in resource-poor settings where ultrasound access is limited.

Clients were counseled on what to expect as side effects and could at any time contact the provider via phone, with vaginal bleeding and abdominal pain most common symptoms. To better manage client expectations, given that 36–41% reported experiencing more bleeding and pain than anticipated, counseling should be revised to emphasize the potential variability in pain and bleeding experiences as this model is expanded. In addition, contrary to our expectations, the variance in pain is significantly different than the variance in bleeding. Thus, these two most reported symptoms should be addressed individually in more detail during counseling. Post-procedure complications were rare when they occurred and they largely could be managed at the health center after additional teleconsultation with the medical doctor. Less than 10% of clients needed to see a provider due to side effects demonstrating that the tested protocol for the management of safe abortion with medication is safe and nurses/midwives can manage the side effects that require additional treatment. The telemedicine model empowered nurses/midwives by connecting them to medical doctors during service provision, allowing for capacity building among these midlevel providers.

Finally, this study found the telemedicine model for abortion services in health centers to be acceptable among Rwandan women. The model not only brought the point of care closer to those who needed services but also employed a patient-centered approach to pregnancy options counseling and abortion care, ultimately improving both perceived and realized quality of care. Thus, enhancing patient satisfaction and clinical outcomes by combining the benefits of in-person care with the convenience and accessibility of telemedicine. In this pilot study, nurses/midwives followed up with the client 3 times which ensured continuous care throughout the abortion process, an opportunity provided by the use of telehealth which likely improved the quality of care received by the client. The telemedicine model in Ghana also had a high level of acceptability with 84% reporting they would opt for the telemedicine service again and 83% stating they would recommend the service [17]. In South Africa, women in the telemedicine group reported nearly a 100% level of satisfaction and women who received telemedicine preferred telemedicine in the future [16]. The positive acceptability and satisfaction ratings among those using various telemedicine abortion care methods point to opportunities for wider implementation in low- and middle-income countries where access to in-person services can be limited.

Telemedicine has the potential to significantly improve equitable access to abortion care, aligning with the preferences of women across various contexts. A recent review of healthcare access in Africa demonstrated the high prevalence of mobile phone usage for information and communication. This highlights the potential of telemedicine to address healthcare access challenges, especially during public health crises [31]. Globally, access to in-person healthcare is hindered by various structural barriers, including geographic isolation [36], inadequate infrastructure [37], financial constraints [38], and a shortage of healthcare providers [39]. Furthermore, accessing safe abortion care is further complicated by social and cultural obstacles such as gender discrimination, stigma, and limited health education [40, 41]. While telemedicine cannot entirely overcome these challenges, it presents a promising avenue for improving access as telecommunications infrastructure continues to expand. Research on telemedicine models specifically for abortion care in low-resource settings is still emerging. Our Rwanda findings and existing evidence [16, 17, 33, 34] support the adaptability of telemedicine models. They can be tailored to various contexts, promoting equitable access to quality abortion services while considering specific needs, resource availability and regulatory requirements.

### Strengths and limitations

The main strength of this study is its novelty with key implications for low-resource settings. Although some evidence for telemedicine in Sub-Saharan Africa exists [16, 17], this study contributes to the evidence-based for settings with legal constraints related to where (health system level) and who (provider level) can provide medication abortion in the first trimester.

The findings from our study should be interpreted with consideration of the following limitations. The results presented are only for those who received services at the health center. Complicated or life-threatening cases may have presented at the hospitals directly, thus outside of the study population. Another limitation is that only clients who answered their phones were able to participate in the client exit interview. In the end, we randomly called and interviewed 50% of those who received medication abortion services at the health center and we do not have records of how many times an individual client was contacted for the interview or why she did not respond. In addition, the random 50% client exit interview cap was arbitrary, so we do not know how significantly different the experiences of those not represented in the sample are from those clients in the sample.

### Policy and program implications

Results from this pilot study resulted in revisions to the abortion clinical guidelines and protocols to include hybrid telemedicine, optimization of task-sharing with mid-level providers via telemedicine in health centers, and maintaining availability of drugs in health centers through changes to pharmacy dispensing mechanisms to include the abortion combination therapy. Since the study ended in January 2022, our research team has focused on translating findings to policies and programs through a series of stakeholder meetings to review research data, develop technical guidelines for programs, and plan the expansion of services to other districts. Following an amended Ministerial Order, medication abortion is now available at health centers. However, it still requires authorization from a medical doctor, either through an in-person visit or a telemedicine consultation. The telemedicine for medication abortion program expansion has started in four additional districts in Rwanda. Additionally, the telemedicine model of service delivery also has the potential to improve access to other quality sexual and reproductive health services. In the future, telemedicine could enable the medical doctor localized at the hospital to help nurses/midwives remotely localized to treat and manage incomplete abortion cases and difficult-to-manage sexual and reproductive health cases.

## Conclusion

This pilot project introduced first-trimester medication abortion in primary-level health centers employing task-sharing to midlevel providers to increase access to safe abortion services. In providing care to a total 242 women over the course of 15 months, this project demonstrated that quality comprehensive abortion care can be provided at health centers in Rwanda with the utilization of telemedicine. Given the demonstrated feasibility, effectiveness, safety and acceptability of telemedicine for first-trimester medication abortion in health centers in Musanze District, the technical guidelines for safe abortion service provision in Rwanda should be updated and this model of service delivery should be scaled up nationally to address inequities in safe abortion access.

## Supplementary Information

Supplementary Material 1

Supplementary Material 2

## Data Availability

The data collected is governed by the Ministry of Health Rwanda and is considered sensitive health information. Due to strict data privacy regulations and ethical considerations, we were unable to obtain the necessary permissions to make this dataset publicly accessible. The data from this study are available from the corresponding author upon reasonable request.
