# Supplementary material for: Innovation through telemedicine to improve medication abortion access in primary health centers: findings from a pilot study in Musanze District, Rwanda
Source: BMC Public Health. 2025 May 7;25:1681. doi: 10.1186/s12889-025-22629-z (PMC12057260; doi:10.1186/s12889-025-22629-z)
Supplement: Supplementary file 1 — Supplementary Material 1 [file 12889_2025_22629_MOESM1_ESM.pdf]

Service Intake Form for Musanze District Telemedicine MA

**IDENTIFICATION**	
101. Patient ID (serial #-HC Code-Patient #-Year) ____	102. Patient Name____________________________
103. District ______________________	
104. Sector _______________________	
105. Cell _________________________	
106. Village ___________________________	
107. Phone Number the woman would prefer to be contacted at for follow-up______________________<br>107a. Second phone number where woman be reached for follow-up_____________________________	
108. Health facility name: ________________________	109.Date the women presented at the HC (first time): ______/_____/______
110. HC nurse-midwife Name: ____	
111. Reasons for the visit (check all that apply)<br>☐ TOP<br>☐ PAC<br>☐ Post-abortion contraception<br>☐ Treatment of side effects<br>☐ Abortion complications<br>Other: __________________________	
112. Did the woman give consent to be contacted in three weeks for the client exit interview conducted by RHIYW?	☐ Yes<br>☐ No

ID

MEDICAL HISTORY

301	Age ☐☐ Years<br>Don't know … 88	302	Education<br>☐ No education<br>☐ Primary<br>☐ Secondary<br>☐ Above secondary	303	Marital Status<br>☐ Never married<br>☐ Married/living together<br>☐ Divorced/Widowed/Separated
304	Time to travel to the health facility<br>☐☐ Hours	305	Mode of transportation to health facility<br>(Check all that apply)		☐ Walk<br>☐ Bus<br>☐ Bicycle<br>☐ Motorbike<br>☐ Car<br>☐ Other: ____________________
		306	How much did you pay for transport?		☐☐

Gynecologic and Obstetric History

307	Gravida ☐☐	308	Parity ☐☐	309	Prior Abortions ☐☐	310	Last Menstrual Period<br>_____/_____/______
311	Number of alive children ☐☐						
312	When did the Last pregnancy before this one end (if ended in live birth, when was last child born?):<br>312a. ☐☐ years ago<br>312b. ☐☐ months ago			313	What was the outcome of last pregnancy before this one (follow up from question 312)?<br>☐ Baby born alive<br>☐ Pregnancy interfered/aborted<br>☐ Miscarriage (spontaneous abortion)<br>☐ Ectopic pregnancy<br>☐ Baby born dead (still birth)<br>☐ Other: ___________________		
314	Use of contraception prior to this current pregnancy:<br>☐ Yes, regularly (always used, method failed)<br>☐ Yes, but not regularly (e.g. forgot to take pills, condoms, etc)<br>☐ No contraceptive use			315	What contraceptive method were you using? (Check all that apply)<br>☐ Condoms<br>☐ Pills<br>☐ Injectables<br>☐ Implant<br>☐ IUD<br>☐ Male sterilization<br>☐ Natural family planning (Period abstinence, etc)<br>☐ Breast feeding<br>☐ Other traditional method<br>☐ None		

ID ☐☐☐☐☐☐☐☐

PHYSICAL EXAMINATION

**VITAL SIGNS**						
401	BP	☐☐☐/☐☐	403	Pulse	☐☐☐ beats/ minute	
404	Respirations per minute	☐☐	405	Temp	☐☐°C	
**Pregnancy Test and Gestational Age**						
406	Pregnancy test result	☐ Not done<br>☐ Positive<br>☐ Negative<br>☐ Inconclusive result				
407	Gestational Age (estimate based on LMP) ☐☐ Weeks					
408	Pelvic Exam ____________________ (Notes)					
409	Ultrasound Exam ____________________ (Notes)					
410	Gestational Age (ultrasound results) ☐☐ Weeks					

		Yes	No	Not observed	Comments
411	Cervix is open	☐	☐	☐	
412	Signs of vaginal infection	☐	☐	☐	Treatment given:
413	Presence of uterine bleeding	☐	☐	☐	☐ Scant ☐ Moderate ☐ Profuse
414	Speculum exam results normal (if no, comment)	☐	☐	☐	

ANCILLARY TESTING

		Yes	No	Not observed	
415	Hemoglobin	☐	☐	☐	
416	Hematocrit/platelets	☐	☐	☐	
417	STI	☐	☐	☐	
418	HIV	☐	☐	☐	
419	Hepatitis B	☐	☐	☐	
420	Hepatitis C	☐	☐	☐	
421	RPR	☐	☐	☐	

ID ☐☐☐☐☐☐☐☐

422	Blood group	☐	☐	☐	☐ A<br>☐ B<br>☐ AB<br>☐ O<br>☐ Don't know
423	RH	☐	☐	☐	☐ Negative<br>☐ Positive<br>☐ Don't know
424	Other tests				
425	Did you see any signs of maltreatment/abuse, risk behaviors or poor mental health status?	☐ Yes<br>☐ No→ Skip to next section			
426	If yes, mark all the apply	☐ Mental illness<br>☐ Signs of extreme anxiety<br>☐ Uncooperative<br>☐ Homelessness<br>☐ Signs of physical abuse →426a<br>☐ Signs of sexual abuse →426a<br>☐ Family rejection<br>☐ Domestic violence →426a<br>☐ Illicit drug use<br>☐ Alcoholism<br>☐ Clinical depress/attempted suicide<br>☐ Other________________________________<br>____			
426a	Did you refer to GBV services?	☐ Yes<br>☐ No			

ID | | | | | | | | |

TELEMEDICINE CONSULTATION

501	Telemedicine visit number	☐ 1<br>☐ 2<br>☐ 3<br>☐ 4<br>☐ 5<br>☐ 6
501a	Date and time of telemedicine visit	Date: __/___/___<br>Time: ________
502	Reason(s) for telemedicine consultation	☐ First consultation for TOP<br>☐ Immediate follow up consultation<br>☐ Management MA of sides effects<br>☐ Management of complications<br>☐ Uncertainty regarding patient care
503	Conclusion from telemedicine consultation (check all that apply)	☐ Woman is pregnant<br>☐ Woman is eligible for TOP at the HC using MA.<br>☐ Woman is referred for TOP at the district hospital<br>☐ Woman prefers services at district hospital<br>☐ Woman prefers surgical abortion; referred to hospital<br>☐ Suspected ectopic pregnancy or abnormal intrauterine pregnancy<br>☐ Woman referred to the hospital for further investigation<br>☐ Uterine anomalies identified<br>☐ Other: ___________________
504	Conclusion from additional teleconsultations (check all the apply)	☐ Reviewed additional exams<br>☐ Prescribed treatment for complications<br>☐ Prescribed additional treatment related to other illnesses<br>☐ Decision to refer<br>☐ Patient wants service at hospital<br>☐ Patient wants surgical abortion<br>☐ Other___________________
505	Comments and notes from clinical examinations: ____<br>____	
506	Post TOP counseling given (check all that apply)	❑ Woman told what she should expect: Bleeding similar to a menstrual period, which may continue up to two (2) weeks, and spotting until the next menstrual period.<br>❑ Woman was informed of possible side effects: pain/cramping; chills/fever; nausea/vomiting; diarrhea.<br>❑ Woman was informed about danger signs to look for after being discharged (heavy bleeding, sign of infection & feeling very sick) and when to contact a medical provider immediately.<br>❑ Woman was advised to come or call immediately (and not to wait) if she has complications or signs of danger

		❑ Contraceptive counseling was given for all methods.<br>❑ Instructions for taking medications, routine hygiene and resuming sexual activity were given.<br>❑ Risk behaviors were described<br>❑ Phone number for provider was given<br>❑ Obtained woman's phone number and appointment for follow up<br>❑ Obtained woman's consent for follow up calls

ID

MEDICATION AND TREATMENT

601	Treatment given<br>☐ Termination of pregnancy<br>☐ Treatment of incomplete abortion	602	Treatment Method	☐ Misoprostol only<br>☐ Mifepristone & Misoprostol<br>☐ MVA<br>☐ D&C<br>☐ Other: ____________________
603	Were there any complications due to the procedure or medication?			☐ Yes<br>☐ No
603a.	Is yes, specify			

Medication administered during treatment

Complete the following section for each of the medications used for treatment of the patient. It includes anesthetics used for MVA; misoprostol; mifepristone; painkillers; anti-emetics, etc.

**Drug** (write name of drug including IV fluids and blood)	**A. Dose** (mg/ mcg/ IU/ mL)	**B. Route of Administration**	**C. Date Taken**	**D. Timing**	**E. Location Taken**
Name					
604. Mifepristone	200 mg	Oral		Record time take at HC	Health Center
605. Misoprostol (1st dose)	800 mcg	Buccal/cheek		24 hours after mifepristone	
606. Misoprostol (2nd dose)	800 mcg	Buccal/cheek		3 hours after 1 dose of miso	
607. Misoprostol (3rd dose) – only administered after telemedicine consultation	800 mcg	Buccal/cheek		As needed	
608. Paracetamol	500 mg	Oral		Every 4 hours as needed	
609.					
610.					
611.					

ID | | | | | | | |

CONTRACEPTIVE COUNSELING & PROVISION		
612	Counseled patient on contraceptive methods	☐ Yes<br>☐ No
613	Contraceptive method provided:	☐ OCP<br>☐ Condoms<br>☐ IUD<br>☐ Injectables<br>☐ Implant<br>☐ Female sterilization<br>☐ Male sterilization<br>☐ Natural family planning methods<br>☐ Referred for preferred method
614	Date contraceptive method provided	_____/_____/_____
615	Condition of patient at discharge	❑ Well<br>❑ Awake, alert/walks unassisted<br>❑ She agrees she is ready to leave<br>❑ Normal vital signs<br>❑ Referred to other facility
616	Date & time of discharge	Date: __/___/___<br>Time: ________
617	Comments	
618	Treatment Done by:	________________________<br>(Name of nurse/midwife)
619	Medical doctor/gynecologist	________________________

ID

FOLLOW-UP VISITS OR CALLS

701	Date of initial visit<br>_____/_______/______ (DD/MM/YY)	702	Date of follow-up Visit<br>_____/_______/______ (DD/MM/YY)
703	Modality used for follow up		☐ Physical follow up visit<br>☐ Phone call
704	Select which follow-up call/visit:		☐ 48 hours<br>☐ 7 days →705<br>☐ 14 days →705<br>☐ Additional follow-up →705
704a	48 hour follow-up (Select all that apply)		☐ Patient took misoprostol properly<br>☐ Patient started to bleed<br>☐ Patient managing side effects well<br>☐ Patient told to come back to HC (Specify)____________ ____________________ _______

Assessment

705	BP ☐☐☐/☐☐	708	Pulse (beats per minute) ☐☐☐
707	Respirations per minute ☐☐	709	Temp ☐☐°C

		Yes	No	Not observed	Comments
710a	Sepsis/infection suspected	☐	☐	☐	
710b	Shock/heavy breathing suspected	☐	☐	☐	
710c	Uterine perforation suspected	☐	☐	☐	

Pelvic Exam & Pregnancy Test

711	Pregnancy test result	☐ Not done ☐ Positive ☐ Negative ☐ Inconclusive result
712	Size of the uterus	☐☐
713	Gestational age	☐☐
714	Pelvic Exam Comments<br>____	

ID

715	Ultrasound Exam Notes				
		Yes (1)	No (2)	Not observed (99)	Comments (96)
716	Cervical is open	☐	☐	☐	
717	Signs of vaginal infection	☐	☐	☐	Treatment given:
718	Presence of uterine bleeding	☐	☐	☐	☐ Scant ☐ Moderate ☐ Profuse
719	Speculum exam results normal (if no, comment)	☐	☐	☐	
720	Result of first procedure:<br>☐ Termination in progress/continuing<br>☐ Treatment success - complete abortion<br>☐ Treatment success - completion of incomplete abortion<br>☐ Lost to follow-up<br>☐ Treatment failure - Incomplete abortion<br>☐ Treatment failure - Continuation of pregnancy<br>☐ Treatment failure - Death of the patient				

Follow-up Treatment Given

722	Treatment given<br>☐ Secondary treatment of incomplete abortion<br>☐ Secondary treatment of continued pregnancy<br>☐ Referral to another facility<br>☐ None – In course<br>☐ None - successful<br>Other: ________________		
723	Treatment Method:<br>☐ Misoprostol alone<br>☐ Mifepristone & misoprostol<br>☐ MVA<br>☐ D&C<br>☐ Other________________________<br>☐ None	724	Treatment given at<br>☐ Same facility as initial visit<br>☐ Another facility (Part of study)<br>☐ Another facility (not part of study)

ID | | | | | | | | |

Symptoms and management during/after treatment<br>(questions to be asked patients in all follow-up visits 7 days and 14 days in person or by phone)					
I am going to read you a list of side effects that may be related to the treatment you have received at the health facility. Can you please tell me if you had any of these side effects after you went home from the health facility (after receiving the treatment). I will also ask you to rate their severity from 1 to 5, if you have experienced some side effects, and some questions about how you managed them:					
**Symptoms**	A. What was the severity on a scale of 1 to 5?	B. Did patient take any medication?	C. Did patient see a health care provider for this side effect?	D. How long did it last approximately?	E. Do patient still have the side effect?
725. Nausea	☐☐<br>Did not have … 99	YES … 1<br>NO … 2	YES … 1<br>(specify)<br>NO … 2	❑ <30<br>❑ 30-60<br>❑ >60	YES … 1<br>NO … 2
726. Vomiting	☐☐<br>Did not have … 99	YES … 1<br>NO … 2	YES … 1<br>NO … 2	❑ <30<br>❑ 30-6<br>❑ >60	YES … 1<br>NO … 2
727. Headache	☐☐<br>Did not have … 99	YES … 1<br>NO … 2	YES … 1<br>NO … 2	❑ <30<br>❑ 30-6<br>❑ >60	YES … 1<br>NO … 2
728. Shivering	☐☐<br>Did not have … 99	YES … 1<br>NO … 2	YES … 1<br>NO … 2	❑ <30<br>❑ 30-6<br>❑ >60	YES … 1<br>NO … 2
729. Vaginal bleeding	☐☐<br>Did not have … 99	YES … 1<br>NO … 2	YES … 1<br>NO … 2	❑ <30<br>❑ 30-6<br>❑ >60	YES … 1<br>NO … 2
730. Abdominal pain	☐☐<br>Did not have … 99	YES … 1<br>NO … 2	YES … 1<br>NO … 2	❑ <30<br>❑ 30-6<br>❑ >60	YES … 1<br>NO … 2
731. Other (specify)<br>________________	☐☐<br>Did not have … 99	YES … 1<br>NO … 2	YES … 1<br>NO … 2	❑ <30<br>❑ 30-6<br>❑ >60	YES … 1<br>NO … 2
732. For a physical follow up consultation:	☐ Minimal or absent bleeding<br>☐ Normal uterine size (small, firm)<br>☐ Uterus and adnexa not tender and no cervical motion tenderness<br>☐ Closed cervical				
733. Note on psychosocial status:	☐ Report doing well /recovered<br>☐ Good Emotional Status<br>☐ Domestic Violence<br>☐ Potential anxiety<br>Specify Other Psychological concerns__________________________				

ID

734.	CONCLUSION FOR FOLLOW UP – CONSULTATION<br>☐ She is doing well/ no danger sign/stay home.<br>o Menstrual-like bleeding and cramping<br>☐ Incomplete evacuation/treatment failure/ woman should come back at health center<br>o Bleeding more than normal menses<br>☐ Present of PAC complications / woman should come back at health center<br>☐ Woman should comeback at the health center for other reasons: specify: ____________________________<br>☐ Woman referred to hospital. Specify reasons; _________________<br>☐ Other conclusion specify: ____________

ID | | | | | | | | |

TREATMENT SUMMARY

201. Venue of TOP
- ❑ District Hospital
- ❑ This Health Center
- ❑ Other________________________

202. Date and Time of Doctor telemedicine consultation	Date: _____/_____/_____ Time: _____________

203. RRH Doctor Name: ________________________________ ____________________________

204. Signature

_____/_____/_____ 205. Date of initial visit (when MA combi pack given)	_____/_____/_____ 206. Follow-up call #1 (48 initial visit)	_____/_____/_____ 207. Follow-up call #2 (7 days later)	_____/_____/_____ 208. Follow-up call #3 (14 days later)

209. Conclusion of 1st tele consultation (Check all that apply)<br>❑ MA Authorized<br>❑ Referred to hospital<br>❑ Contraceptive method adopted first visit<br>❑ Need to return for contraceptive method<br>❑ Other______________________	210.Treatment prescribed:<br>☐ Misoprostol only<br>☐ Mifepristone + Misoprostol<br>☐ Other:<br>________________	211. Modality agreed upon with woman for 1st follow-up call/visit:<br>☐ Cell phone<br>☐ Physical consultation

212. Outcome for the 1st follow up consultation (7 days):
- ☐ Woman doing well; no danger sign, woman told to stay home.
- ☐ Treatment failed
- ☐ Woman told to come back at the health center for physical examination
- ☐ Woman referred to hospital. Specify reasons: _________________
- ☐ Other outcome: _________________

213. Outcome for 2nd follow up consultation (14 days):
- ☐ Treatment successful
- ☐ Treatment failed
- ☐ Woman told to come back to the health center for physical examination
- ☐ Woman referred to hospital
  Reasons: _______________________
- ☐ Other: _________________

214. Condition of patient at discharge
- ❑ Well
- ❑ Referred to other facility
