# Supplementary material for: Innovation through telemedicine to improve medication abortion access in primary health centers: findings from a pilot study in Musanze District, Rwanda
Source: BMC Public Health. 2025 May 7;25:1681. doi: 10.1186/s12889-025-22629-z (PMC12057260; doi:10.1186/s12889-025-22629-z)
Supplement: Supplementary file 2 — Supplementary Material 2 [file 12889_2025_22629_MOESM2_ESM.pdf]

## *Client Exit interviews for Musanze District Telemedicine MA*

### *Ibibazo bibazwa uwamaze guhabwa servisi - Gukoresha Ikorana buhanga murwego rwo gutanga servisi zihereranye nu ubuzima bw' imyororokere*

*Instructions: (RHIYW Researchers) Use this form to interview each consenting woman 2-4 weeks after she received medication abortion services at health center. Responses should be collected in Qualtrics. All instructions for the interviewer are written in italics.*

***Amabwiriza: (Umushakashatsi ukora muri RHIYW) akoresha ibi bibazo kubaza umugore/umukobwa waherewe servisi/imiti ku kigo nderabuzima.ibisubizo byinjizwa muri Qualtrics. Amabwiriza yose aheraranye nuko ibazwa riri bugende yandikwa muri italics.***

**Name of Facility:** ________________________________ **Today's Date:** __ __ / __ __ / __ __ __ __
*Izina ry'ikigo nderabuzima* *Itariki*

***INTERVIEWER, PLEASE READ:***
*Hello, my name is __________, and I am a researcher with Rwanda Health Initiative for Youth and Women. As you remember, I am calling because you have agreed that a member of our team will contact you for a follow-up call 2-3 weeks, the time you were receiving services at the health center. I am following up with you for the second part of the research study that you consented to enroll in at the time of receiving PAC services. I would like to ask you questions about your recent experiences with PAC and telemedicine services. We are collecting this information in order to improve these services. I will not write down your name on the data collection form. Everything you tell me will be kept strictly confidential. No one will be able to identify you from the information we collect. Your participation is voluntary, and you do not have to answer questions that you do not want to answer. It should take about 30 minutes to answer the questions. Do I have your permission to continue?*

***Muraho Nitwa ______________________nturuka muri RHIYW. Nkuko mubyibuka, ndaguhamagara kuko wemeye ko umwe mubagize itsinda ryacu ry'ubushakashati azaguhamagara nyuma y'ibyumweru 2-3 igihe wakiraga serivisi ku kigo nderabuzima kugirango akurikirane uko servisi wahawe zagenze nuko Umerewe. Nguhamagaye murwego nk'igice cya cya kabiri cy'ubushakashatsi; Turimo gukusanya makuru kugirango tunoze serivisi. Ntabwo nzandika izina ryawe kurupapuro rwo gukusanya amakuru. Ibyo umbwiye byose bizabikwa ibanga rwose. Ntamuntu numwe uzashobora kukumenya mumakuru dukusanya. Uruhare rwawe ni ubushake, kandi ntutegetswe gusubiza ibibazo udashaka gusubiza. Birafata iminota 30 kugirango usubize ibibazo. Ese mumpaye uruhushya rwo gukomeza?***

**Participant Verbal Consent[1]:**

---

[1] Written consent was obtained during enrollment in the study at the time when services were received. Consenting women agreed to have their data included in the study and also to be contacted for a follow-up call 2-4 weeks after services were received.

I understand the purpose of this interview. I have had the opportunity to ask questions about this interview.

______I agree to participate

______I do not wish to be interviewed

Date: __ __ / __ __ / __ __

**Kwemera kwitabira ubushakashatsi muburyo bw'amagambo**

Kwemera kwitabira ubushakashatsi muburyo bwanditse, umugo/umukobwa yabutanze mugihe yahabwaga servisi ku gigo nderabuzima. Abagore bemeye ko amakuru yabo ashyirwa mubushakashatsi kandi bakanemerwa kuzahamagarwa nyuma y'ibyumweru 2-4 serivisi barahawe servisi.

Ndemeza ko numva intego y'iki kiganiro. Kandi ko Nagize amahirwe yo kubaza ibibazo bijyanye niki kiganiro.

**Itariki:-----/-----/-----**

****Before continuing with the questions, ensure the woman is in a location with auditory privacy.***

******Mbere yo gukomeza nibibazo, menya neza ko umugore ari ahantu hamwe n'ibanga ryumva.*

| Section 1: Abortion/PAC experience<br>Igika cya1: Uko imiti yamumereye | | |
|---|---|---|
| **Q6** | How did you learn about medication abortion services at health facility?<br><br>Wamenye ute amakuru y'uko servisi zihereranye no gukuramo inda hakoreshejwe imiti ziboneka ku kigo nderabuzima? | ❑ Walk-in/Self-referral<br>❑ Community health worker referral<br>❑ Health post referral<br>❑ Youth center referral<br>❑ Police/RIB<br>❑ Community leaders<br>❑ Other private health facility<br>❑ Other (Specify) ______________<br>❑ Not Applicable<br><br>▪ Nijye wijyanye<br>▪ Noherejwe n'umujyanama w'ubuzima<br>▪ Nohejwe na post de sante<br>▪ Noherejwe ni ikigo cy'urubyiruko<br>▪ Na Police/RIB<br>▪ N'abayobozi b'inego z'ibanze<br>▪ N'ibigo ndebuzima byigenga<br>▪ Ibindi(bivuge)_______________<br>▪ Ntabwo bihuye |
| **Q7** | What services did you receive at the HC | Treatment of incomplete abortion<br>Termination of pregnancy →Q9 |
| **Q8** | Do you know that medication abortion is now available at some health centers? | Yes →Q10<br>No→Q10 |
| **Q9** | What was your reason for requesting TOP? | Child<br>Rape<br>Forced marriage<br>Incest<br>Health of women/fetus |
| **Q10** | How many days has it been since you took your misoprostol tablets?<br>Hashize iminsi ingahe ufashe ibinini bya | Number of days: ____<br>Umubare w'iminsi:______ |

| | | |
|---|---|---|
| | misoprostol? | |
| **Q11** | Did anything make self-care/ taking your misoprostol at home complicated for you?<br>Ese hari ikigeze kikugora mubihereranye no kwiyitaho murugo, ndetse no gufata imiti ya misoprostol murugo? | Yes (Describe what)<br>No<br>I took misoprostol at the health center<br>▪ Yego<br>▪ Oya |
| **Q12** | Did you have pain after you took the misoprostol tablets?<br>Ese wigeze ufata imiti yo kukugabanyiriza ububabare nyuma yo gufata ibinini bya misoprostolo? | ❑ Yes<br>❑ No ***(Skip to question Q14)***<br>❑ Don't Know/Refused<br>▪ Yego<br>▪ Oya<br>▪ simbizi |
| **Q13** | [If YES] Would you describe the pain as mild, moderate, or severe?<br><br>Niba aribyo. Watubwira urugero rw'ububabare wagize niba buringaniye cyangwa niba ububabare wagize bwari bukabije cyane? | ❑ Mild<br>❑ Moderate<br>❑ Severe<br>❑ Not Applicable<br>▪ Buke<br>▪ Buringaniye<br>▪ Bukabije<br>▪ ntibihuye |
| **Q14** | Did you have heavy bleeding after taking the pills?<br>Ese wigeze uva cyane nyuma yo gufata umuti? | ❑ Yes<br>❑ No ***(Skip to question Q16)***<br>❑ Don't Know/Refused<br>▪ Yego<br>▪ Oya<br>▪ simbizi |
| **Q15** | [If YES] Did you require to go to the health center for additional care?<br>Niba ari yego, Byagisabye gusubira ku kigo nderabuzima? | ❑ Yes<br>❑ No<br>▪ Yego<br>▪ Oya |
| **Q16** | Did you see any tissue come out of your vagina between the time you took the pills and now?<br>Wigeze ubona ibintu bimeze nk'inyama biva mu gitsina cyawe guhera igihe wafatiye ibinini kugeza ubungubu? | ❑ Yes<br>❑ No<br>❑ Don't Know/Refused<br>▪ Yego<br>▪ Oya<br>▪ Simbizi |
| **Q17** | Were you satisfied with your abortion/TOP/PAC procedure?<br>Ese wunvise unyuzwe nuburyo bakoresheje bagukuriramo inda? | ❑ Yes<br>❑ No<br>❑ Don't Know/Refused<br>▪ Yego<br>▪ Oya<br>▪ Simbizi |
| **Q18** | How did you feel about the telemedicine consultation?<br><br>Wumvise umeze ute igihe bagusuzumaga, muvugana na muganga hakoreshejwe mudasobwa? | ❑ Did not need a telemedicine consultation<br>❑ Acceptable<br>❑ Too long<br>❑ Embarrassed/uncomfortable<br>❑ Don't know/refused<br>❑ Other (specify)<br>▪ Bimeze neza/mbyishimiye<br>▪ Ari birebire<br>▪ Biteye ubwoba/ntabyishimiye<br>▪ ntibimworoheye<br>▪ simbizi/sinshaka kubivugaho<br>▪ Ibindi (vuga) |
| **Q19** | In total, how many times did you go to the health facility for abortion/PAC services from the time you first knew you were pregnant until now?<br><br>Muri rusange, ni inshuro zingahe wagiye ku kigo nderabuzima gushaka serivisi zo gukuramo inda kuva igihe wamenyeye ko utwite kugeza ubu? | ______times<br><br>_________Inshuro |
| **Q20** | How prepared did you feel for what happened during this abortion/PAC?<br>Wari witeguye gute ibyakubayeho mugihe cyo gukuramo inda? | ❑ Very prepared ***(Skip to Q22)***<br>❑ Somewhat prepared<br>❑ Not at all prepared<br>❑ Don't know/Refused |

| | | |
|---|---|---|
| | | ▪ Narimbyiteguye cyane<br>▪ Nari mbyiteguye murugero<br>▪ Ntabwo nari mbiteguye na gato<br>▪ Simbizi |
| **Q21** | ***[If somewhat or not at all prepared**:]*<br>Tell us what were you not prepared for or just somewhat prepared for regarding abortion/PAC? Which aspects or side effects of the procedure were you not prepared during consultation?<br>*Niba wari ubyiteguye murugero cyangwa utari ubyiteguye*<br>Tubwire ibyo utari witeguye cyangwa ibyo wari witeguye murugero kubyerekeye gukuramo inda?<br><br>Nibihe bintu cyangwa izihe ngaruka zakubayeho utari witeguye mugihe cyo kugusuzuma ? | ❑ Pain<br>❑ More bleeding than expected<br>❑ Vomiting<br>❑ Shivers<br>❑ Fever<br>❑ Don't know/Refused<br>❑ Other (describe)<br><br>▪ Kubabara<br>▪ Kuva cyane<br>▪ Kuruka<br>▪ Gutengurwa<br>▪ Kugira umulilo<br>▪ Simbizi<br>▪ Ibindi:_______________ |
| **Q22** | Did you take any pain medication during your abortion/PAC?<br>Hari imiti wafashe igabanya uburibwe mugiye wakuragamo inda? | ❑ Yes<br>❑ No ***(Skip to Q24)***<br>❑ Don't Know/Refused<br>▪ Yego<br>▪ Oya<br>▪ Simbizi |
| **Q23** | Was the pain medication you took enough to reduce your pain to a comfortable level?<br>Ese imiti wafashe ikugabanyiriza uburibwe yaragufashije nezakuburyo wunva umerewe neza? | ❑ Yes<br>❑ No<br>❑ Don't Know/Refused<br>Yego<br>Oya<br>Simbizi |
| **Q24** | Did you take any additional medication during TOP/PAC?<br><br>Ese hari undi muti wafashe igihe bagukuriragamo Inda? | ❑ Yes<br>❑ No ***(Skip to Q26)***<br>❑ Don't Know/Refused<br>▪ Yego<br>▪ Oya<br>▪ simbizi |
| **Q25** | If yes, which additional medication did you take?<br><br>Niba hari indi miti wafashe niyihe? | ❑ Antibiotics<br>❑ Traditional medicine<br>Other specify:________________<br>Antibiyotike<br>Imiti ya Kinyarwanda<br>Iyindi(yivuge):_____________ |
| **Q26** | Have you spoke to the health provider since you began the TOP/PAC procedure?<br><br>Wigeze uterefona umuforomo/umuganga ukora aho ariho hose kuva wafatiye ibinini bya misoprostol (cyangwa ibinini bya kabiri) murugo? | ❑ Yes, I called the nurse for follow-up consultation<br>Yes, the nurse called me for follow up consultation<br>❑ ***No, I did not have phone follow-up consultation(Skip to Q28)***<br>❑<br><br>▪ Yego<br>▪ Oya<br>▪ |
| **Q27** | Did you experience any technical issues during the phone call appointment with the health providers?<br><br>Wigeze uhura nikibazo cya tekiniki mugihe cyo guhamagara/yangwa kuvugana kuri terefone numuforomokazi/umuganga? | ❑ I felt comfortable talking to the nurse over the phone.<br>❑ Phone was not accessible to me<br>❑ It was easy to hear<br>❑ Whenever I called the nurse-midwife I managed to talk to her<br>❑ I had difficulties reaching the nurse-midwife on the phone<br>❑ It was difficult for me to find the cell phone to call<br>Other<br>▪ Nunvise aribyiza kandi ntakibazo kuvugana nu |

| | | |
|---|---|---|
| | | umugoromokazi kuri telephone<br>▪ Sinari pfite Telefoni<br>▪ Ntabwo namwunvaganeza<br>▪ Igihe naguganye nu umuforomokazi nunvaga neza<br>▪ Nagize ikibazo cyo sinavugisha umuforomokazi kuri telefoni<br>▪ Byarangoye cyane kubona telefoni muvugishirizaho |
| **Q28** | Have you gone to see a health provider since you began the TOP/PAC procedure?<br><br>Wigezeujya kureba undimuforomokazi/umuganga ahandi ahariho hose nyuma yo gufata ibinini bya Misoprostol murugo? | ❑ Yes<br>❑ No ***(Skip to Q33)***<br>❑Don't Know/Refused<br>▪ Yego<br>▪ Oya<br>▪ simbizi |
| **Q29** | If YES, Where did you go?<br><br>Niba hari aho wagiye kureba undi muganga tubwire ni he? | ❑ I came back to this facility<br>❑ Public hospital<br>❑ Public health Center<br>❑ Pharmacy<br>❑ Other private health facility<br>❑ Traditional healers<br>❑ Community health worker<br>❑ Other (Specify) _______________<br>❑ Not Applicable<br>▪ Nagarutse kuriki kigo nderabuzima<br>▪ Ku bitaro<br>▪ Kukigo nderabuzima<br>▪ Kuri farumasi<br>▪ Ikigo nderabuzima kigenda<br>▪ Kubaganga ba gihanga<br>▪ Ku umujyanama w'ubuzima<br>▪ Ahandi<br>▪ Simbizi:________________________ |
| **Q30** | If YES, Why did you go to see the health provider?<br><br>Niba waragiyeyo niyihe mpanvu yakujyanyeyo? | ❑ More than two weeks of bleeding<br>❑ Bleeding more than normal menstrual bleeding<br>❑ Severe or increased pain<br>❑ Fever<br>❑ Chills<br>Phone call consultation/follow-up was not possible for me<br>Provided asked me to return to HC<br>❑ Other (Specify) ___________<br>❑ Not Applicable<br>▪ Nari ndengeje ibyumweri bi biri nkiva<br>▪ Naravaga cyane kuruta uko nva iyondi mumihango<br>▪ Ububabare bwari bukaije/ bwariyongeraga cyane<br>▪ Nahindaga umulilo<br>▪ Naratengurwaga<br>▪ Ibindi;_______________ |
| **Q31** | If YES, Can you tell me what happened there?<br>Niba hari aho wagiye kureba undi muganga mbwira uko byagenze<br><br>***Probe: Did they do a surgical abortion with MVA or D&C? Did you have to go into the theater? Did you stay overnight?***<br>***Ibibazo ushobora kubaza: Bigeze bakuvurisha ubundi buryo nkubwa MVA, cg D&C? wigeze ujya kubagwa?wigeze urara mu bitaro?*** | ______________________<br>______________________<br>______________________<br><br>❑ Not Applicable<br>▪ Ntabwo bijyanye |

| | | |
|---|---|---|
| **Q32** | If YES, did the doctor or nurse tell you if you were still pregnant?<br>Niba aribyo , umuganga yaba yarakubwiyeko inda yari ikirimo? | ❑ Yes<br>❑ No<br>❑ Don't Know/Refused<br>❑ Not Applicable<br>▪ Yego<br>▪ Oya<br>▪ Simbizi<br>▪ ntibihuye |
| **Q33** | During the follow up the follow-up calls at 7 and 14 days after TOP/PAC visit, did the provider ask you if you had any questions?<br><br>Igihe umuforomo yaguterefonaga nyuma yuko uhabwa servisi iminsi 7 hamwe na 14, yigeze akubaza niba hari ikibazo ufite? | ❑ Yes<br>❑ No<br>❑ Don't Know/Refused<br>▪ Yego<br>▪ Oya<br>▪ Simbizi |
| **Q34** | Do you have any unanswered questions now about your procedure or your care?<br><br>Hari ikibazo ugifite ubu kitasubijwe gihereranye n'uburyo cyangwa na servisi? | ❑ Yes<br>❑ No<br>❑ Don't Know/Refused<br>▪ Yego<br>▪ Oya<br>▪ Simbizi |
| **Q35** | If yes, what else would you want to know?<br>Niba gihari , ese ni ikihe kindi wifuza kumenya? | |

| **Section 2: Family Planning** | | |
|---|---|---|
| **Q36** | Did the provider talk to you about family planning during the first visit for TOP/PAC?<br>Umuforomo/umuganga yigeze akubwira kubihereranye no kuboneza urubyaro mugihe wasurwaga bwa mbere? | ❑ Yes<br>❑ No ***(Skip to Q38)***<br>❑ Don't Know/Refused<br>▪ Yego<br>▪ Oya<br>▪ Simbizi |
| **Q37** | If yes, did you take a method home or were instructed to come back after the last TOP/PAC?<br>Niba ari yego hari uburyo bwo kuboneza urubyaro wigeze utahana murugo cyangwa bagusabye ko uzagaruka kubufata nyuma yuko ufata ibinini byanyuma? | ❑ Yes took method home ***(Skip Q41)***<br>❑ Yes was instructed to come back for a method<br>❑ No ***(Skip Q39)***<br>❑ Don't Know/Refused<br>▪ Yego natahanye uburyo murugo<br>▪ Yego bansabye ko nzagaruka gufata uburyo<br>▪ Oya<br>▪ simbizi |
| **Q38** | Did you return to the health facility for a family planning method?<br><br>Wasubiye ku kigo nderabuzima gufata uburyo bwo kuboneza rubyaro? | ❑ Yes ***(Skip to Q40)***<br>❑ No<br>❑ Don't Know/Refused<br><br>▪ Yego<br>▪ Oya<br>▪ Simbizi |
| **Q39** | Did you talk to someone else outside of the health facility personnel about family planning during your TOP/PAC?<br>Wigeze urugana nundi muntu utari uwo kukigo nderabuzima kubihereranye no kuboneza urubyaro nyuma yo gukuramo Inda? | ❑ Yes Pharmacist<br>❑ Yes CHW<br>❑ Yes Other (describe)<br>❑ No ***(Skip to Q42)***<br>❑ Don't Know/Refused<br>▪ Yego ukora murifarumasi<br>▪ Yego Umujyanama<br>▪ Yego undi<br>▪ Ntawe<br>▪ simbizi |

| | | |
|---|---|---|
| **Q40** | Did you adopt a family planning method<br>Wafashe uburyo bwo kuboneza urubyaro? | ❑ Yes<br>❑ No ***(Skip to Q42)***<br>❑ Don't Know/Refused<br>▪ Yego<br>▪ Oya<br>▪ Simbizi/narabyanze |
| **Q41** | Which method did you adopt?<br><br>N'ubuhe buryo wafashe? | ❑ Oral contraceptive pills<br>❑ Injectable contraceptives<br>❑ Implant<br>❑ IUD<br>❑ Condoms<br>❑ Other (Specify)<br>***[All responses skip to next section]***<br><br>▪ ***Uburyo bw'ibinini***<br>▪ ***Uburyo bw'urushinge***<br>▪ ***Agapira***<br>▪ ***Ubryo bwa agapira ko mumula***<br>▪ ***Udukingirizo***<br>▪ ***Ubundi (buvuge)*** |
| **Q42** | If you did not adopt family planning, why you did not adopt it<br><br>Niba ntaburyo wafashe , nukubera iki utabufashe? | ❑ I choose to not take birth control<br>❑ I will use natural methods<br>❑ I do not need FP since I am not sexually active, it was just because I was raped<br>❑ I decided to take it later<br>❑ Other_______________________________________<br><br>▪ Nahisemo kudafata uburyo<br>▪ Nzakoresha uburyo bwa kamere<br>▪ Ntabwo nkeneye uburyo wbo kuboneza kuko ntabwo mubusanzwe nkora imibonano mpuza bitsina nuko gusa nari narafashwe kungufu<br>▪ Nahisemo kuzabufata ubutaha<br>▪ Ibindi_____________________________ |

| **Section 3. Quality of Care** | | |
|---|---|---|
| **Q43** | Do you feel that the facility staff treated you respectfully when you initially requested care?<br><br>Wunva abakozi bi ikigo nderabuzima barakwakiriye neza igihe watangiraga gusaba servisi? | ❑ Yes<br>❑ No<br>❑ Don't know/Refused<br>Yego<br>Oya<br>Simbizi/simbishaka |
| **Q44** | Were the information and explanations regarding the procedures you received from the health providers adequate?<br><br>Amakuru n'Ibisobanuro wahawe bihereranye nu uburyo bwo gukuramo inda wunvise bihagije? | ❑ Yes<br>❑ No<br>❑ Don't Know/Refused<br>▪ Yego<br>▪ Oya<br>▪ Simbizi/simbishaka |
| **Q45** | Did the provider ask if you have any pain, itching, discharge or sores in or around your vagina?<br>Ese umuganga yakubajije niba ufite uburibwe,gutengurwa, nuko ibintu biva mu gitsina bimeze? | ❑ Yes<br>❑ No<br>❑ Don't Know/Refused<br>▪ Yego<br>▪ Oya<br>▪ Simbizi/simbishaka |

| | | |
|---|---|---|
| **Q46** | Did the health provider tell you what warning signs you should look for after leaving the facility that mean that you should go to the nearest health center or hospital right away?<br><br>Ese umuganga yakubwiye ibimenyetso mpuruza ugomba kwigengeseraho ,wabigira ukihutira kujya kukigo nderabuzima cyangwa ibitaro kekwegereye ako kanya? | ❑ Yes<br>❑ No<br>❑ Don't Know/Refused<br>▪ Yego<br>▪ Oya<br>▪ Simbizi/simbishaka |
| **Q47** | Were you given a phone number to call if you needed help or had any questions or concerns after you left the facility?<br><br>Ese Baguhaye numero ya telefoni uzahamagara niba ukeneye ubundi bufasha cyangwa ufite ikibazo igihe watashye uvuye kukigo nderabuzima? | ❑ Yes<br>❑ No<br>❑ Don't Know/Refused<br>▪ Yego<br>▪ Oya<br>▪ Simbizi/simbishaka |
| **Q48** | Did you call or visit the health center after the first visit when you received TOP/PAC?<br>Wigeze uhamagara kukigo nderabuzima nyuma yuko bamaze kugukuriramo inda? | ❑ Yes I called<br>❑ Yes I visited<br>❑ Did not need to call or visit<br>❑ Don't Know/Refused<br>▪ Yego narahamagaye<br>▪ Yego nasubiyeyo<br>▪ Sinahamagaye cyangwa ngo nsubirey0<br>▪ Simbizi/nabyanze |
| **Q49** | When did you call or visit the health provider?<br>Niryari wahamagaye cyangwa wasubiye kukigo nderabuzima? | ❑ Before 7 days<br>❑ Between 7-14 days<br>❑ Don't Know/Refused<br>▪ Mbere y'iminsi 7<br>▪ Hagati y'iminsi 7 na 14<br>▪ Simbizi/simbisubiza |
| **Q50** | How would you rate the care you received during this call?<br>Ibiheraranye no kuvugana na muganga kuri telefoni wavugako byagenze gute? | ❑ Excellent<br>❑ Good<br>❑ Fair<br>❑ Poor<br>❑ Don't know/Refused<br>▪ Neza cyane<br>▪ Neza<br>▪ Murugero<br>▪ Nabi<br>▪ Simbizi/simbishaka |
| **Q51** | Did the doctors or nurses tell you to be looking for the following warning signs at home that mean you should go to the nearest health center or hospital right away? (These are sometimes called "complications").<br>Ese muganga /umufromokazi yakubwiye kwitondera ibimenyetso mpuruza murugo, bivugako uramutse ubigize ukwiye kwihutira kugera ku kigo nderabuzima caygwa ibitaro Bikwegereye?<br><br>*Interviewer read list and mark all that apply.* | ❑ More than two weeks of bleeding<br>❑ Bleeding more than normal menstrual bleeding<br>❑ Severe or increased pain<br>❑ Fever<br>❑ Chills<br>❑ Other (Specify) __________<br>▪ Kuva birengeje ibyumweru bibiri<br>▪ Kuva byinshi birengeje imihango<br>▪ Kubabara cyane cyangwa ububabare bwiyongera<br>▪ Guhinda Umulilo |
| **Q52** | Did the health provider tell you about the need to avoid sex until a few days after bleeding stops?<br>Ese umuforomo yakubwiyeko uzaba uretse gukora imibonano mpuzabitsina mu minsi mike ikurikira nyuma yuko Kuva bihagarara? | ▪ ❑ Yes<br>❑ No<br>❑ Don't Know/Refused<br>▪ Yego<br>▪ Oya<br>▪ Simbizi/simbishaka |
| **Q53** | Did the health care provider tell you that without using a contraceptive method you could get pregnant again quickly, even before your next menstruation?<br><br>Ese umuforomo/muganga yakubwiyeko nudakoresha uburyo bwo kubineza urubyaro ushobora kongera gutwita vuba, nambere yuko wongera kujya mumihango? | ❑ Yes<br>❑ No<br>❑ Don't Know/Refused<br>▪ Yego<br>▪ Oya<br>▪ Simbizi/simbishaka |

| | | |
|---|---|---|
| **Q54** | Did the heath provider assure you that the information that you shared would be kept private?<br>Umuganga cyanga umuformokazi yakubwiye ko amakuru yose wamuhaye azabikwa muburyo bwibanga? | ❑ Yes<br>❑ No<br>❑ Don't Know/Refused<br>▪ Yego<br>▪ Oya<br>▪ Simbizi/simbishaka |
| **Q55** | Was the health provider courteous and polite?<br>Ese umuganga/umuforomo yakwakiriye muburyo bwiza? | ❑ Yes<br>❑ No<br>❑ Don't Know/Refused<br>▪ Yego<br>▪ Oya<br>▪ Simbizi/simbishaka |
| **Q56** | Do you feel you can trust your health care provider to keep your information private?<br>Ese wunva wizeye umuganga/umuforomo ko azakugirira ibanga/ko amakuru yawe atazayasakaza? | ❑ Yes<br>❑ No<br>❑ Don't Know/Refused<br>▪ Yego<br>▪ Oya<br>▪ Simbizi/simbishaka |
| **Q57** | Did you feel you had enough privacy while being treated at this facility?<br>Ese wunva baraguhaye servisi muburyo bw'ibanga? | ❑ Yes<br>❑ No<br>❑ Don't Know/Refused<br>▪ Yego<br>▪ Oya<br>▪ Simbizi/simbishaka |
| **Q58** | If you ever needed an abortion again would you choose this method?<br><br>Ese biramutse bikubayeho ko wongera gukenera servisi zo gukuramo Inda, wakitamo ubu buryo bwakoreshejwe? | ❑ Yes<br>❑ No<br>❑ Don't Know/Refused<br>▪ Yego<br>▪ Oya<br>▪ Simbizi/simbishaka |
| **Q59** | Would you recommend that a friend or family member wanting a medication abortion come to this facility for care?<br><br>Ese wagira inama inshuti yawe yifuza servisi zo gukuramo inda kugana iki kigo nderabuzima? | ❑ Yes *(Skip to question Q61)*<br>❑ No<br>❑ Don't Know/Refused<br>• Yego<br>• Oya<br>• Simbizi/simbishaka |
| **Q60** | **IF NO**, why not?<br><br>Niba ari oya, nukubera iki? | ❑ Facility is too far from home<br>❑ Providers were unkind/rude<br>❑ No female provider was available<br>❑ Procedure was too expensive<br>❑ Waiting time was too long<br>❑ Facility seems poorly maintained (dirty; no running water; infrastructure concerns)<br>❑ Other (specify) ________<br>❑ Not Applicable<br><br>• Iki kigo nderabuzima kiri kure yahoo dutuye<br>• Abaganga ntabwo bapfashe neza<br>• Ntabwo navuwe numugore kandi aribyo nifuzaga<br>• Byari bihenze cyane guhabwa servisi<br>• Nategereje igihe kirekire<br>• Ikigo nderabuzima nta suku gifite, nibyumba bakoreramo sibyiza<br>• Indi mpanvu(sobanura):____________<br>• Ntibihuye:_______________ |

| | | |
|---|---|---|
| **Q61** | Overall, how would you rate the care you received during your first visit at the health facility for TOP/PAC?<br><br>Muri rusange tubwire uko ubona servisi waherewe kukigo nderabuzima igihe wasuzumagwa bwa mbere kugirango bagukuriremo inda? | ❑ Excellent<br>❑ Good<br>❑ Fair<br>❑ Poor<br>❑ Don't know/Refused<br>• Byiza cyane<br>• Byiza<br>• Biringaniye<br>• Bibi<br>• Simbizi/simbishaka |
| **Q62** | How would you rate the care you received during the first follow-up call at 7 days after TOP/PAC procedure?<br><br>Muri rusange tubwire uko ubona uko ubona ikiganiro mwagiranye na muganga iminsi 7 nyuma yo guhabwa servisi? | ❑ Excellent<br>❑ Good<br>❑ Fair<br>❑ Poor<br>❑ Don't know/Refused<br><br>• Byiza cyane<br>• Byiza<br>• Biringaniye<br>• Bibi<br>• Simbizi/simbishaka |
| **Q63** | How would you rate the care you received during the second follow-up call at 14 days after TOP/PAC procedure?<br><br>Muri rusange tubwire uko ubona uko ubona ikiganiro mwagiranye na muganga iminsi 14 nyuma yo guhabwa servisi? | ❑ Excellent<br>❑ Good<br>❑ Fair<br>❑ Poor<br>❑ Don't know/Refused<br><br>• Byiza cyane<br>• Byiza<br>• Biringaniye<br>• Bibi<br>• Simbizi/simbishaka |
| **Q64** | Do you have suggestions about how we could have made this experience better or easier for you?<br><br>Ese hari ibyifuzo ufite bihereranye nuko servisi wahawe n'uburyo bakwitayeho byakosorwa/byarushaho kugenda neza? | ______________________________________<br><br>______________________________________ |

| **Section 4. Socio-Demographic Information**<br>**Ibibazo bihereranye ni imibereho rusange** | | |
|---|---|---|
| **Q65** | How old are you?<br>Ufite imyaka ingahe?<br><br>***If client is under 20, be sure to complete Section 6: Youth Supplement Form at the end of the survey.*** | _____ years<br><br>_______Imyaka |
| **Q66** | What is your highest level of education?<br><br>Amashuri wize ni ayahe? | ❑ No education<br>❑ Some primary school<br>❑ Completed primary school<br>❑ Some secondary school<br>❑ Completed secondary school<br>❑More than secondary<br>❑ Don't know/Refused<br>• Sinigeze niga<br>• Nageze mu mashuli abanza<br>• Narangije amashuri abanza<br>• Nageze muyisumbuye<br>• Narangije ayisumbuye |

| | | |
|---|---|---|
| | | • Nize arenga ayisumbuye<br>• simbizi |
| **Q67** | Are you currently in school?<br>Ese ubu uri umunyeshuri? | ❑ Yes<br>❑ No<br>• Yego<br>• Oya |
| **Q68** | What is your relationship status?<br><br>***Interviewer: Please read all answers and mark the one that best describes the interviewee's relationship status***<br><br>**Ese imibanire yawe ni iyihe?** | ❑ Married<br>❑ Single got pregnancy from violence<br>❑ Living with partner, but not married<br>❑ Have a steady partner, but not living together<br>❑ Separated/Divorced<br>❑ Widowed<br>❑ No steady partner<br>❑ Other (Specify) ________________________<br>• Narashatse<br>• Ndi ingaragu nuko nafashwe kungufu<br>• Mbana n'umugabo nuko tutashakanye byemewe namategeko<br>• Nfite umugabo nubwo tutabana<br>• Natandukanye nu umugabo<br>• Ndi umupfakazi<br>• Ntamugabo uhoraho ngira<br>• Ibindi(bivuge):___________________ |
| **Q69** | How many times have you become pregnant? (Include all pregnancies, including this one.)<br><br>Ni ishuro yakangaye utwita (vuga zose harimo niyingiyi wari utwite)? | ____ pregnancies<br><br>_________inda |
| **Q70** | How many children do you have living with you now?<br>Ufite abana bangahe babana nawe ubungubu? | ____ children<br>*(If zero, skip to question 208)*<br>____*abana* |
| **Q71** | How old is your youngest child?<br>Umwana wawe muto afite imyaka ingahe? | ____ years<br>❑ Not Applicable<br>_________Imyaka |
| **Q72** | Do you currently do any kind of work that you get paid for?<br>Hari akazi ubungubu ufite kaguhemba? | ❑ Yes<br>❑ No<br>• Yego<br>• oya |
| **Q73** | How far do you live from this health facility?<br><br>Gereranga intera iri hagati yahoo utuye naho ikigo nderabuzima Kiri? | ▪ A short walking distance away<br>❑ More than 5 kilometers<br>❑ More than 10 kilometers<br>❑ Outside of this town/city/outsides the sector where the health center is localized<br>▪ Intera ni ngufi<br>▪ Intera iri hejuru y'ibirometero 5<br>▪ Intera iri hejuru y'ibirometero 10<br>▪ Ni hanze y'umurenge ntuyemo |

## Section 5. Youth Supplement Form

*Complete this section for **women under 20** only*

*Aha huzuzwa ku urubyiruko gusa*

| | | |
|---|---|---|
| **Q74** | *For interviewer only: Is the client under 24 years of age?*<br>*Ese uwahawe servisi afite imyaka iri munsi ya 24?* | ❑ Yes<br>❑ No *(Survey complete)*<br>*Yego*<br>*oya* |
| **Q75** | Did you feel that you were treated differently at the health facility because you are young?<br>Ese wigeze ubona bakwakiriye muburyo butanoze kuko ukiri muto/uri urubyiruko? | ❑ Yes<br>❑ No<br>❑ Don't Know/Refused<br>Yego<br>Oya<br>simbizi |
| **Q76** | Did you feel that you were treated differently at the health facility because you are unmarried?<br>Ese wigeze ubona bakwakiriye muburyo butanoze kuko utarashaka? | ❑ Yes<br>❑ No<br>❑ I am married<br>❑ Don't Know/Refused<br>▪ Yego<br>▪ Oya<br>▪ Narashatse<br>▪ Simbizi/ndifashe |
| **Q77** | Did you feel that you were treated differently at the health facility because requesting abortion services?<br>Ese wigeze ubona bakwakiriye muburyo butanoze kuko ushaka servisi yo gukuramo inda? | ❑ Yes<br>❑ No<br>❑ Don't Know/Refused<br>▪ Yego<br>▪ Oya<br>▪ Simbizi |
| **Q78** | Did the facility require your parents' consent before treating you?<br>Ese abaganga bo ku kigo nderabuzima baba barasabye ko ababyeyi bawe bakwemerera kugirango uhabwe servisi? | ❑ Yes<br>❑ No<br>❑ Don't Know/Refused<br>Yego<br>Oya<br>Simbizi |
| **Q79** | Did the facility require your consent before treating you?<br>Ese abaganga bo ku kigo nderabuzima baba baragusabye ko wowe ubwawe ubemerera kugirango baguhe servisi? | ❑ Yes<br>❑ No<br>❑ Don't Know/Refused<br>▪ Yego<br>▪ Oya<br>▪ simbizi |
